# Evaluating the Combined Effectiveness of Influenza Control Strategies and Human Preventive Behavior

**DOI:** 10.1371/journal.pone.0024706

**Published:** 2011-10-17

**Authors:** Liang Mao

**Affiliations:** Department of Geography, University of Florida, Gainesville, Florida, United States of America; Umeå University, Sweden

## Abstract

Control strategies enforced by health agencies are a major type of practice to contain influenza outbreaks. Another type of practice is the voluntary preventive behavior of individuals, such as receiving vaccination, taking antiviral drugs, and wearing face masks. These two types of practices take effects concurrently in influenza containment, but little attention has been paid to their combined effectiveness. This article estimates this combined effectiveness using established simulation models in the urbanized area of Buffalo, NY, USA. Three control strategies are investigated, including: Targeted Antiviral Prophylaxis (TAP), workplace/school closure, community travel restriction, as well as the combination of the three. All control strategies are simulated with and without regard to individual preventive behavior, and the resulting effectiveness are compared. The simulation outcomes suggest that weaker control strategies could suffice to contain influenza epidemics, because individuals voluntarily adopt preventive behavior, rendering these weaker strategies more effective than would otherwise have been expected. The preventive behavior of individuals could save medical resources for control strategies and avoid unnecessary socio-economic interruptions. This research adds a human behavioral dimension into the simulation of control strategies and offers new insights into disease containment. Health policy makers are recommended to review current control strategies and comprehend preventive behavior patterns of local populations before making decisions on influenza containment.

## Introduction

During the past decade, influenza has obtained unprecedented attention due to widespread occurrence of novel viruses, such as the bird flu in 2003 and the swine flu in 2009 [1], [2]. Recent estimates by the Center of Disease Control and Prevention (CDC) indicated that the 2009 swine flu is responsible for 274,000 hospitalizations and 12,470 deaths in the United States [3]. These staggering health burdens call for effective measures to control and prevent future outbreaks. The control of influenza primarily involves applying health resources to affected people, known as control strategies, for example, medical treatment for infected individuals, closure of affected workplaces/schools, and travel restriction to affected communities [4]. The prevention of influenza emphasizes healthy people and depends on their voluntary behavior against the disease, referred to as the preventive behavior. As recommended by CDC, the preventive behavior against influenza include receiving vaccination, wearing facemasks, washing hands frequently, taking antiviral drugs, and others [5].

While devising various control strategies and evaluating their effectiveness, few studies have incorporated the preventive behavior of individuals [6], [7]. In most cases, individuals are often assumed to passively comply with control strategies, but their active prevention against the disease has been overlooked. In reality, the preventive behavior of individuals also reduces infections and takes effect concurrently with typical control strategies. For instance, individuals may voluntarily protect themselves from infection, once they realize some control strategies being applied to their family members, colleagues, or communities [8], [9], [10]. By far, the combined effectiveness of control strategies and individuals' preventive behavior remains unclear, and little attention has been paid to this issue. Lack of such knowledge may bias estimation of health resources needed to suppress an outbreak, and mislead the real practice of influenza containment.

The purpose of this article is to evaluate the combined effectiveness of control strategies and individual preventive behavior. Agent-based stochastic simulations are used to investigate three control strategies, including the Targeted Antiviral Prophylaxis (TAP), workplace/school closure, and travel restriction, as well as combinations of all three. The urbanized area of Buffalo, New York, USA, is taken as a study area. The control effectiveness with and without considering individual preventive behavior is compared to indicate if there exists a significant difference. Cost-effective strategies are suggested based on the comparison analysis. The remainder of this article is organized as follows. The method section that follows reviews two established influenza models for simulation and describes the design of control strategies being simulated. The result section presents and compares the simulation results. The discussion section concludes this article with implications.

## Materials and Methods

Epidemic models, including mathematical and computer models, have been extensively used to investigate disease control strategies, because of their ease and flexibility to deal with different scenarios. The classic mathematical model, the SIR model, and its variants employ differential equations to describe continuous variations between three subpopulations, i.e., the susceptible, infectious and recovered [11], [12]. Various control strategies are often expressed as different initial conditions (e.g., the size of susceptible population) or parameter settings (e.g., the infection rate) of differential equations. The computer-based simulation models have recently gained their impetus in epidemiology [13], [14], [15], [16]. These models study population-level health outcomes through the simulation of individuals and their micro-interactions. Control strategies can be represented by altering individuals' health status and their behavior, such as endowing them immunity against infection and prohibiting their out-of-home activities. All of these epidemic models provide solid platforms to evaluate and compare alternative strategies, thus informing health policy making [17], [18].

### Epidemic models with and without individual preventive behavior

Epidemic models without considering individual preventive behavior are widely seen in the literature and hereinafter referred to as ‘influenza-only’ models, because they primarily focus on influenza transmission. In this research, an influenza-only model is implemented in the study area, which includes a total number of 985,001 individuals. These individuals live in 967 census block groups and 400,870 households according to US census 2000 [19], and carry out daily activities in 36,839 business locations [20]. The model involves an agent-based stochastic simulation, discrete time steps, and spatially explicit representation of individuals. Each individual is a modeling unit with a set of characteristics (e.g., age, occupation, infection status, location and time of daily activities) and behaviors (e.g., traveling between locations for activities and having contact with other individuals) [21], [22]. Individuals and households are simulated under the constraints of census data so that the modeled population matches the age and household structure of the real study area. Individuals are also assigned to business locations to represent their daily activities, such as working, shopping, eating out, etc. (Figure S1). The contacts between individuals take place when individuals meet at the same time and location, such as homes, workplaces, shops, and restaurants. Because individuals travel over time and location, their mobility weaves a spatio-temporally varying contact network (See Text S1 Section 1.1). Through such a network, influenza viruses diffuse from one individual to another. Each individual is allowed to take one of four infection status during a time period, i.e. susceptible, latent, infectious, and recovered. The progress of infection status follows the natural history of influenza, including the latent, incubation, and infectious periods (Table S1). During the infectious period, individuals may manifest symptoms and become symptomatic. To initiate the disease transmission, five infectious individuals are randomly seeded into the study area at the first day of simulation, which then lasts for 150 days. In each day, the model traces susceptible contacts of infectious individuals, and stochastically identifies the next generation of infections using the Monte-Carlo method (See Text S1 Section 1.2).

In order to further consider individual preventive behavior, this research employs an agent-based ‘dual-diffusion’ stochastic model that simulates the concurrent diffusion of both influenza and individual preventive behavior [23]. The preventive behavior is considered as a practice or information that also diffuses over contact networks through inter-personal influence. These two diffusion processes interact with one another, i.e., the diffusion of influenza motivates the propagation of preventive behavior, which in turn limits the influenza diffusion [24], [25], [26]. In the model, the diffusion of influenza is simulated similarly to the influenza-only model aforementioned. The diffusion of individual preventive behavior is propelled by two types of inter-personal influence through the contact network: one is the perceived infection risk and the other is the perceived social standard. The former is represented as the proportion of influenza cases among an individual's contacts, while the latter is expressed as the proportion of behavioral adopters among the contacts [23]. Individuals are simulated to evaluate these two proportions every day through the contact network. Once either proportion exceeds a corresponding threshold, an individual will be convinced to adopt and practice preventive behavior [27], [28]. The estimation of individualized thresholds toward adoption is based on a health behavioral survey approved by the Social and Behavioral Sciences Institutional Review Board, University at Buffalo, State University of New York. The waiver of informed consent was obtained from the university review board for this research (See Text S1 Section 2 and Figures S2–S3). Compared to the influenza-only model, individuals in the dual-diffusion model have additional characteristics, such as their adoption status of preventive behavior and thresholds toward adoption. Individuals also have more behaviors, for example, evaluating infection risks and social standards from their contacts, making decision to adopt, and carrying out preventive behavior against influenza. For illustrative purposes, the use of flu antiviral drugs (e.g., Tamiflu) is taken as an example of preventive behavior in the simulation, because its clinical efficacy is more conclusive than other behaviors, for instance, washing hand and wearing facemasks. Specifically, if an individual uses antiviral drugs, the chance of being infected and infecting others can be reduced by 70% and 40%, respectively [14], [29]. Implementation details of these two models are not the focus of this article, and readers could refer to Text S1 Section 1.3 and Table S2.

### Influenza control strategies

Influenza control strategies are mostly applied at three levels: the individual level, group level, and community level. For each level, one strategy is selected for subsequent investigation, namely, a Targeted Antiviral Prophylaxis (TAP) strategy at the individual level, a workplace closure strategy at the group level, and a travel restriction strategy at the community level, as shown in Table 1. Detailed descriptions of the three strategies are provided below.

**Table 1:** Design and simulation of control scenarios.

Epidemic ModelsStrategies	Influenza-only model(without preventive behavior, PB)	Dual-diffusion model(with preventive behavior)
**Baseline scenario**	No control strategiesNo preventive behavior	N/A
**#1: TAP**	Low: 60% cases	Low: 60% cases+PB
	High: 80% cases	High: 80% cases+PB
**#2: School/workplace closure (WC)**	Low: 100% schools+10% workplaces	Low: 100% schools+10% workplaces+PB
	High:100% schools+33% workplaces	High: 100% schools+33% workplaces+PB
**#3: Travel restriction (TR)**	Low: 10% trips	Low: 10% trips+PB
	High: 50% trips	High: 50% trips+PB
**#1+#2+#3**	Low: combined by all “lows” above	Low: combined by all “lows” above+PB
	High: combined by all “highs” above	High: combined by all “highs” above+PB

First, the TAP strategy identifies symptomatic individuals (influenza cases), searches their household members, and then targets antiviral drugs to all these individuals [13], [30]. This strategy has been recommended to be quite effective if stockpiles of antiviral drugs are sufficient and infections can be quickly detected [4]. To account for limited health personnel, this research assumes that only a proportion of influenza cases, 60% (60%TAP) and 80% (80%TAP), can be identified during a day, following the design by Germann et al. [13]. Second, the workplace closure strategy shuts down a proportion of workplaces/schools where influenza cases are identified [31]. This strategy has been suggested to be useful to socially distance individuals, delay the disease spread, and win time for developing vaccines and antiviral drugs [32]. Following the work by Ferguson et al. [33], a low-level scenario (10%WC) closes 10% affected workplaces and 100% affected schools during a day, while a high-level scenario (33%WC) closes 33% affected workplaces and 100% affected schools. Third, the travel restriction strategy aims to reduce the trips into and out of affected communities [33], [34]. Each of the 967 census block groups in the study area is treated as a community. Following the project by Germann et al. [13], a low-level scenario (10% TR) restricts 10% trips into and out of all affected communities, while a high-level scenario prohibits 50% trips (50% TR). In addition to testing the three control strategies individually, the combinations of all three are also evaluated. A low-level combination scenario (referred to as the Combined-Low) includes all three strategies at their respective low levels. Likewise, a high-level combination (referred to as Combined-High) contains all three single strategies at high levels.

The three control strategies and their combinations in Table 1 are simulated by the influenza-only model and the dual-diffusion model, respectively. Results from the influenza-only model indicate the effectiveness of control strategies without individual preventive behavior. Meanwhile, outcomes from the dual-diffusion model show the combined effectiveness of both control strategies and individual preventive behavior. These two modeled effectiveness are compared to a baseline epidemic scenario, which represents a worst situation of no control strategies and no preventive behavior. All strategies are assumed to be implemented at the time when the cumulative number of influenza cases exceeds 1,000 (1‰ of the population), and last until the end of the epidemic. Individuals having or having not adopted preventive behavior are treated the same by all control strategies, so that the control effectiveness from two models are comparable.

**Figure 1:**
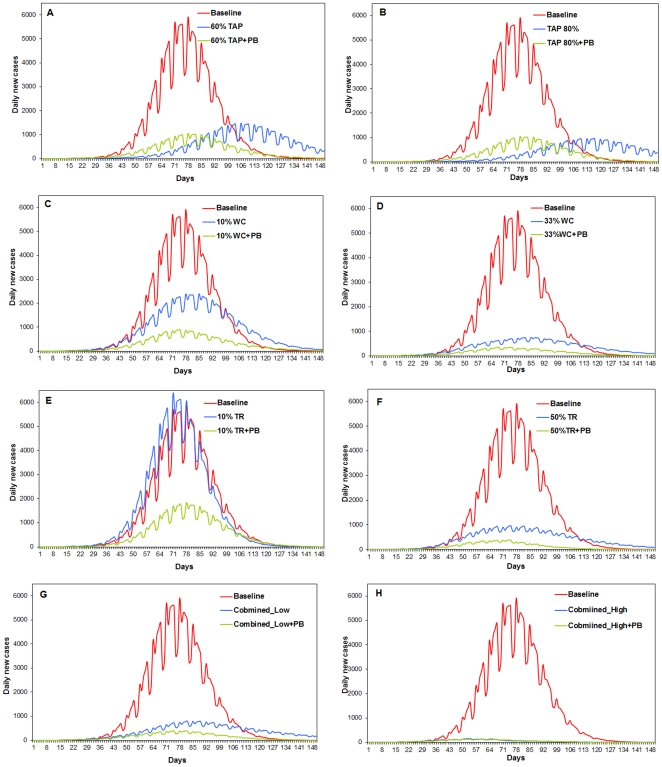
Simulated epidemic curves resulting from control scenarios with/without considering preventive behavior (PB). The curve depicts the number of daily new influenza cases during the course of an epidemic. (A) 60%TAP; (B) 80% TAP; (C) 10% WC; (D) 33% WC; (E) 10% TR; (F) 50% TR; (G) Combined-Low; (H) Combined-High.

For each model and each strategy scenario in Table 1, the simulation is performed 50 realizations to reduce randomness, resulting in a total of 1,000 realizations (5 strategies×2 scenarios×2 models×50 realizations). Each simulation records the time and location of every infection event during a 150-day period. For each strategy scenario, the control effectiveness is measured by an epidemic curve that depicts the number of daily new influenza cases from Day 1 to Day 150. The number of daily new cases is averaged from 50 model realizations, and then plotted against time to form an averaged epidemic curve (Figure 1). Associated characteristics of this epidemic curve are also derived, including an overall attack rate (the percentage of influenza cases in the population) and epidemic peak time (Table 2). For the ease of comparison, a relative effectiveness of a control strategy is also calculated as an index ranging from 0 to 1. The relative effectiveness is defined as a ratio of the attack rate reduced by a strategy from the baseline to the baseline attack rate, i.e., (Baseline attack rate−Attack rate under a strategy)/Baseline attack rate. A zero value represents the baseline scenario without any control strategy (the attack rate under non-strategy = the baseline rate), while a higher value close to 1 indicates that a control strategy produces a smaller attack rate. An effective strategy is expected to produce a low epidemic curve, small attack rate, and high relative effectiveness. In this research, an epidemic is assumed to be successfully contained, if the overall attack rate is below 5%. This is because reported influenza epidemics often have a 5% or higher attack rate [35], [36].

**Table 2:** Control effectiveness of scenarios with/without preventive behavior (PB).

Scenarios	Overall attack rate (%)	Epidemic peak time (Days)	Relative Effectiveness^b^
Baseline	18.60 [18.52, 18.74]^a^	77 [64, 92]	0.00
60% TAP	6.87 [0.00, 8.89]	90 [3,136]	0.63
80% TAP	4.74 [0.00, 7.49]	82 [3,145]	0.75
60% TAP+PB	4.31 [0.00, 5.20]	71 [3,104]	0.77
80% TAP+PB	4.30 [0.00, 4.96]	76 [5,102]	0.77
10% WC	11.87 [11.36, 11.90]	80 [64,71]	0.36
33% WC	4.86 [0.00, 5.42]	69 [4, 94]	0.74
10% WC+PB	3.95 [0.00, 4.99]	66 [3, 98]	0.79
33% WC+PB	1.83 [0.00, 2.46]	61 [3, 103]	0.90
10% TR	20.00 [19.91,20.11]	74 [64, 85]	−0.07
50% TR	5.91 [0.00, 6.61]	65 [6, 89]	0.68
10% TR+PB	7.10 [0.00, 8.70]	67 [3, 97]	0.62
50% TR+PB	1.65 [0.00, 2.11]	60 [3, 89]	0.91
Combined Low	5.00 [4.33, 5.73]	86 [72, 103]	0.73
Combined High	0.72 [0.00, 0.94]	60 [5, 108]	0.96
Combined Low+PB	1.95 [0.32, 2.34]	75 [7, 108]	0.90
Combined High+PB	0.68 [0.00, 0.91]	52 [4, 102]	0.96

a All measures are the averages of 50 model runs, and 95% confidence intervals are shown in brackets.

b Relative effectiveness = (Baseline attack rate- Attack rate under a strategy)/Baseline attack rate.

T-test shows that the relative effectiveness with and without PB is significantly different (*p*-value = 0.043).

**Figure 2:**
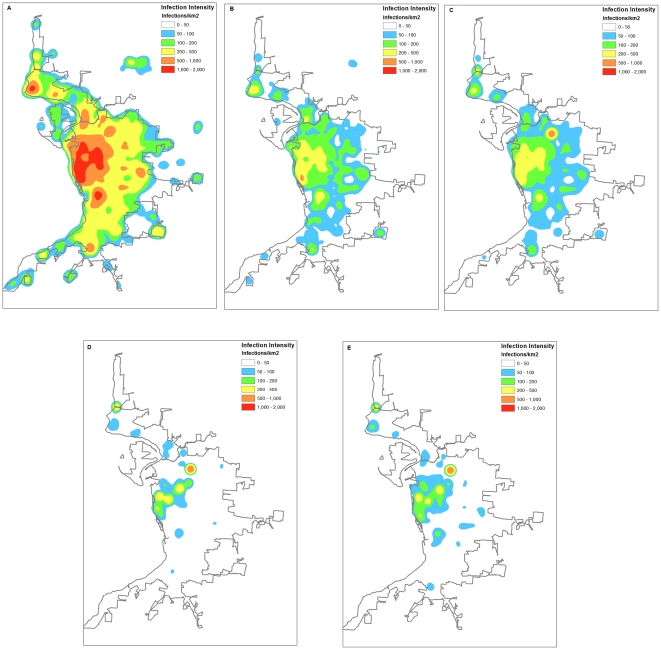
Intensity maps of cumulative infections for the entire epidemic. (A) Baseline scenario, (B) 60% TAP+PB, (C) 10% WC+PB, (D) 50% TR+PB, and (E) Combined Low+PB. The color ramp represents the 150-day cumulative number of infections per sq km^2^ at a 50 m×50 m cell location. The infection intensity is further categorized into 6 levels, i.e., very low (0–50 infections/km^2^), low (50–100), moderate (100–200), high (200–500), very high (500–1,000), and extremely high (>1,000).

The spatial effectiveness of control strategies is also of interest, and thus a series of infection intensity maps are displayed in Figure 2. The infection intensity represents the density of total infections as points occurring within every geographic unit (50 m×50 m) during the entire 150-day epidemic. The intensity value at each cell location is also the average from 50 model realizations and is converted to a unit of infections per sq km^2^ for the ease of comparison. An effective strategy is expected to reduce infection intensity at every location, and meanwhile confine the spatial extent of affected areas.

## Results

### Targeted Antiviral Prophylaxis (TAP) strategy at the individual level

On average, the baseline epidemic scenario (red curves in Figure 1) causes an 18.6% of the population developing influenza symptoms (Table 2). The epidemic peaks at Day 77 with approximately 6,000 new cases occurring at the peak time. The application of 60% TAP and 80% TAP scenario (blue curves in Figure 1A–B) significantly reduces the overall attack rate to 6.87% and 4.74%, respectively. These two TAP scenarios also postpone the peak time by 5–13 days. Without considering preventive behavior, the 80% TAP scenario seems effective to contain the epidemic, because it manages to lessen the overall attack rate under the 5% epidemic criterion.

By further adding the preventive behavior (hereinafter abbreviated as PB), both 60%TAP+PB and 80%TAP+PB scenarios (green curves in Figure 1A–B) result in even lower attack rates around 4.3% (Table 2). The epidemic peaks can be limited around 1,000 daily new cases, while the peak time remains similar to the baseline scenario. This is because the diffusion of preventive behavior quickly exhausts the pool of susceptible individuals, and thus fewer individuals can be infected. The results indicate that if the preventive behavior of individuals is considered, both the 60% TAP+PB and 80% TAP+PB achieve a similar control effectiveness ( = 0.77), leading to mild attack rates that may not qualify as an epidemic. The 60% TAP, rather than the 80% TAP, would be sufficient enough to contain the epidemic. Health agencies only need to prepare a smaller stockpile of antiviral drugs than would otherwise being expected.

### Workplace closure (WC) strategy at the group level

Turning to the workplace closure strategies (blue curve in Figure 1C), the 10% WC scenario slightly reduces the overall attack rates to 11.87%, and delays the peak time only a little (Table 2). In contrast, the 33% WC scenario ((blue curve in Figure 1D) lessens the attack rate to a much lower level of 4.86%, and advances the peak time by approximately 1 week. For the purpose of containing the epidemic, the 33% WC scenario, i.e., the closure of 33% affected workplaces, is needed to achieve an attack rate under 5%.

By further including the individual preventive behavior (green curves in Figure 1C–D), the 10% WC+PB and 33%WC+PB scenarios produce a much smaller attack rate of 3.99% and 1.83%, respectively (Table 2). The time to reach epidemic peaks is shortened to 61–66 days, roughly 2 weeks earlier than the baseline scenario. The relative effectiveness of 10% WC scenario is doubled by considering preventive behavior. A primary reason is that a number of susceptible individuals voluntary protect themselves from infection. These individuals, therefore, cannot be infected or infect others at workplaces and schools, largely limiting the disease transmission. The comparison suggests that given the preventive behavior of individuals is counted, a 10% workplace closure strategy, instead of the 33% one, would be adequate to contain an influenza epidemic.

### Travel restriction (TR) at the community level

Surprisingly, the 10% TR scenario (Figure 1E) alone causes an even worse situation than the baseline scenario. The overall attack rate reaches 20%, and is 1.4% higher than the baseline rate, leading to a negative effectiveness ( = −0.07 in Table 2). A possible reason is that the travel restriction strategy extends the time of individuals spent at home, thereby intensifying the within-home transmission. Since only 10% of trips into and out of affected communities are restricted, the disease can still be easily transported from one affected community to another through the 90% unrestricted trips. The epidemic thus develops faster and affects more individuals. As the travel restriction level elevates to 50% (the 50% TR in Figure 1F), much more trips into and out of affected communities are restricted. Although the infections at homes are intensified, most infections can only take place within communities, instead of between communities. As a result, the overall attack rate drops to 5.91% and the epidemic peak is greatly mitigated. Nevertheless, the 50% TR does not suffice to contain the epidemic, because the attack rate remains above 5%.

The simulation results are distinctly different if adding individual preventive behavior (green curves in Figure 1E–F). The 10%TR+PB scenario produces a much better outcome than that from the 10% TR alone, because the relative effectiveness jumps from −0.07 to 0.62. The overall attack rate and epidemic peak size are remarkably reduced, although the attack rate remains above 5% (Table 2). The 50%TR+PB scenario turns out to be effective for influenza containment, because the overall attack rate can be lowered to 1.65%, much less than the 5% epidemic criterion.

### Combined control strategies

The combined strategies (the blue curves in Figure 1G–H) outperform each of the three single strategies. The total infections can be contained far below 5% of the population, with a small peak size under 1,000 cases. Particularly, the Combined-High scenario is capable of preventing the epidemic, given only 0.68% of the population being infected (Table 2). Among the three single strategies, the TAP strategy reduces infections within households, the workplace closure strategy tends to prevent infections at workplaces, and the travel restriction limits the disease transmission between communities. These three single strategies work together as complements, leading to a significant improvement in control effectiveness (relative effectiveness>0.9). Without considering preventive behavior, the Combined-High scenario seems necessary to contain the epidemic, while the Combined-Low scenario is insufficient. This argument, however, may be changed by incorporating individual preventive behavior (green curves in Figure 1G–H). The Combined-Low+PB scenario now is adequate to reduce the overall attack rate below 5% and thus contain the epidemic, while the high-level scenario is no longer a necessity.

### Spatial effectiveness of control strategies and preventive behavior

Based on the comparison analysis above, the TAP 60%+PB, 10% WC+PB, 50% TR+PB, and the Combined-Low+PB scenarios are suggested to be cost-effective in controlling influenza epidemics. Therefore, their spatial effectiveness is further examined and compared through infection intensity maps (Figure 2). For description purposes, the mapped infection intensity is further categorized into 6 levels, i.e., very low (0–50 infections/km^2^), low (50–100), moderate (100–200), high (200–500), very high (500–1,000), and extremely high (>1,000).

The baseline scenario (Figure 2A) induces an extremely high intensity of infections in the central business district of the study area. The infection intensity decreases in an outward direction to suburbs. This is because the central business district has the densest residential population and highly concentrated business locations. Compared to the baseline map, the 60% TAP+PB scenario (Figure 2B) greatly reduces the infection intensities all around the study area, although the central business district retains a high-level intensity. The spatial effectiveness of 10%WC+PB (Figure 2C) is similar to the 60% TAP+PB, but moderate infections are more scattered in the suburban areas. The 50% TR+PB is capable of confining the wide spread of influenza over the study area (Figure 2D), leaving only a small number of separated areas with high infection intensity. These hotspots are most located within CBD, university campuses, and large industrial plants, where a large number of people work and live. This is probably because the city-wide travels of individuals are partially prohibited, and hence disease can only develop locally. Finally, the Combined low+PB scenario not only reduces the intensity of the infections at all locations, but also confines spatial extent of disease spread (Figure 2E). The infections in the central business district are reduced to a moderate level, while a vast proportion of the study area has only a small number of infections.

## Discussion

In summary, previous studies on influenza containment have only considered the effectiveness of applying control strategies, while overlooking the effectiveness from individuals' preventive behavior. This research estimates the combined effectiveness of both control strategies and individual preventive behavior. The results imply that previous studies on control strategies are incomplete, and the control effectiveness might be under-estimated. The comparison between two model results indicates that preventive behavior of individuals has an extra effectiveness, in addition to the effectiveness from typical control strategies alone. This extra effectiveness produces an even smaller attack rate of influenza, lower epidemic peak, and earlier peak time. By considering the combined effectiveness, the control of influenza epidemics may not require as much health resources as estimated in previous studies. For example, the 80% TAP strategy could be replaced by the 60% one, reducing the burden of local agencies to prepare health resources. Likewise, the 10% workplace closure strategy, rather than the 33% strategy, would be sufficient to control the seasonal influenza epidemic in the study area. Enormous socio-economic disruptions could be possibly avoided. A low-level combination of the three strategies is recommended to suppress influenza epidemic in the study area, while a high-level combination is no longer a must. Particularly, with the help of individual preventive behavior, the 50% travel restriction strategy and the low-level combined strategy can successfully confines the spatial dispersion of influenza in the study area.

Similar to any modeling analysis, this research has a number of limitations. First, the simulation models focus on one US metropolitan area, one influenza virus strain, and one preventive behavior. It is possible that the model outcomes vary in different cities and different disease parameters, such as a pandemic influenza virus. The interpretation of model outcomes should be limited to seasonal influenza and in the study area. Although the use of antiviral drugs is taken as an example in this research, the methodology can be easily extended to other preventive behavior, such as washing hands and wearing facemasks, once their preventive efficacy is conclusively quantified. Second, the mass media also influences people's decision to adopt preventive behavior, especially for diseases that are highly infectious or pose severe health risks, such as the severe acute respiratory syndrome (SARS). This research has not modeled the mass media because its effects on flu-related preventive behavior remain inconclusive. In addition, the seasonal influenza simulated in this research has a relatively mild infectivity and limited risks, thus is usually not a focus of mass media attention. Third, the model assumes that individuals adopt preventive behavior immediately after the threshold effects happen. In reality, individuals' adoption of a behavior may take a relatively longer period as it may involve a number of psychological steps [37]. A more sophisticated behavioral approach may improve the modeling reality, but also increase the complexity of model structure. A trade-off between model performance and detail levels is always a challenge for modelers [38]. Ongoing research is intended to address these limitations and challenges.

Control strategies enforced by health agencies and preventive behavior voluntarily practiced by the public are two intertwined components of disease containment. Ignoring either component may prevent us from effectively mitigating burdens of influenza on public health. It is hard to resist citing and rephrasing the argument by Funk et al. [26] that “individual self-initiated behavior can change the fate of an outbreak, and its combined effectiveness with control strategies requires proper understanding if we are to fully comprehend how these control measures work”. This research attempts to fuse the human behavioral dimension into the study of control strategies, and thus offers more comprehensive understandings on disease containment. Health agencies are recommended to gain prior knowledge about behavioral patterns of local people before choosing influenza control strategies. The findings of this research call for a review of current control strategies and re-estimate the health resources that are necessary to contain epidemics. It is believed that such a review would shed new insights on improving control effectiveness for looming influenza pandemics.

## Supporting Information

Figure S1 **The simulation of contact network.** The assignment of individuals to households, workplaces, service places and neighbor households based on the attribute and spatial information of individuals.
(TIF)

Figure S2 **Estimated distribution of the threshold of infection risks by gender.** The *X* axis indicates the proportion of influenza cases in the contacts of a participant that is needed to convince the participant to adopt. The *Y* axis shows the frequency of such proportion occurring in the survey results.
(TIF)

Figure S3 **Estimated threshold distribution of adoption pressure by gender.** The *X* axis indicates the proportion of adopters in the contacts of a participant that is needed to convince the participant to adopt. The *Y* axis shows the frequency of such proportion occurring in the survey results.
(TIF)

Table S1 **Model parameters for simulating influenza.**
(DOCX)

Table S2 **Model parameters for simulating preventive behavior.**
(DOCX)

Text S1 (DOC)
