# Supplementary figures and images for: Evaluating the Combined Effectiveness of Influenza Control Strategies and Human Preventive Behavior
Source: PLoS One. 2011 Oct 17;6(10):e24706. doi: 10.1371/journal.pone.0024706 (PMC3197180; doi:10.1371/journal.pone.0024706)

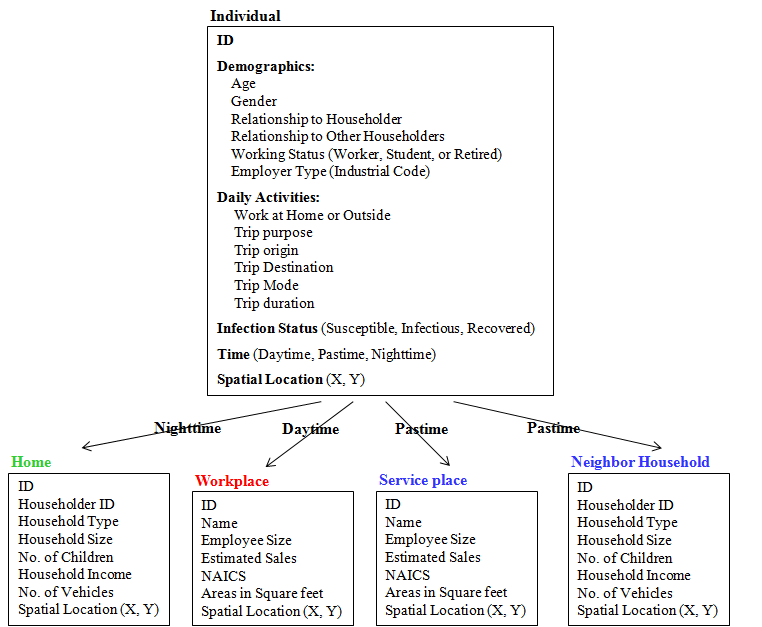

Supplement: Figure S1 — The simulation of contact network. The assignment of individuals to households, workplaces, service places and neighbor households based on the attribute and spatial information of individuals. (TIF) [file pone.0024706.s001.tif]

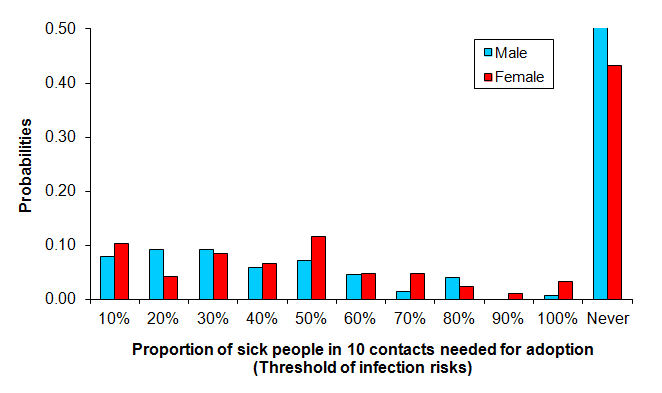

Supplement: Figure S2 — Estimated distribution of the threshold of infection risks by gender. The X axis indicates the proportion of influenza cases in the contacts of a participant that is needed to convince the participant to adopt. The Y axis shows the frequency of such proportion occurring in the survey results. (TIF) [file pone.0024706.s002.tif]

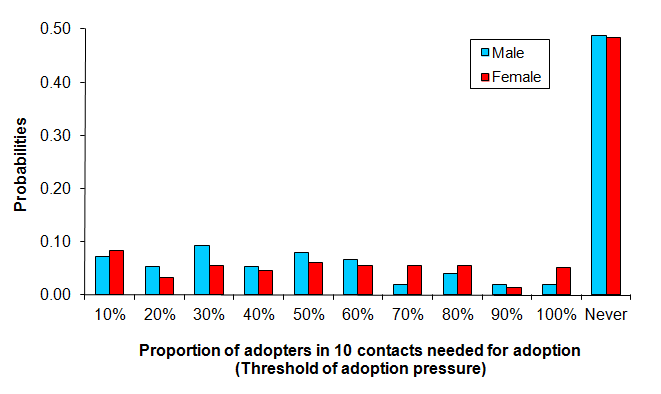

Supplement: Figure S3 — Estimated threshold distribution of adoption pressure by gender. The X axis indicates the proportion of adopters in the contacts of a participant that is needed to convince the participant to adopt. The Y axis shows the frequency of such proportion occurring in the survey results. (TIF) [file pone.0024706.s003.tif]
