# Supplementary material for: Evaluating the Combined Effectiveness of Influenza Control Strategies and Human Preventive Behavior
Source: PLoS One. 2011 Oct 17;6(10):e24706. doi: 10.1371/journal.pone.0024706 (PMC3197180; doi:10.1371/journal.pone.0024706)
Supplement: Table S1 — Model parameters for simulating influenza. (DOCX) [file pone.0024706.s004.docx]

**Table S1** Model parameters for simulating influenza*

| **Parameters** | **Values** | **Literature** |
| --- | --- | --- |
| Transmissibility (R_0_) | 1.3-1.4 | [[6](#_ENREF_6),[7](#_ENREF_7)] |
| Probability of receiving infection | Children: 0.10  Youth and Adults: 0.08  Senior: 0.09 | Calibrated based on R_0_ |
| Latent period | 2 days on average | [[8](#_ENREF_8)] |
| Incubation period | 3 days on average | [[8](#_ENREF_8),[9](#_ENREF_9)] |
| Infectious period | 4-7 days dependent on age groups | [[8](#_ENREF_8),[10](#_ENREF_10)] |
| Likelihood of developing symptoms after infection | 50% | [[11](#_ENREF_11)] |

*. The table is adopted from previoius work in [[1](#_ENREF_1)]
