# Supplementary material for: Evaluating the Combined Effectiveness of Influenza Control Strategies and Human Preventive Behavior
Source: PLoS One. 2011 Oct 17;6(10):e24706. doi: 10.1371/journal.pone.0024706 (PMC3197180; doi:10.1371/journal.pone.0024706)
Supplement: Table S2 — Model parameters for simulating preventive behavior. (DOCX) [file pone.0024706.s005.docx]

**Table S2** Model parameters for simulating preventive behavior^*^

| Reduction in the likelihood of being infected | 70% | [[15](#_ENREF_15),[16](#_ENREF_16)] |
| --- | --- | --- |
| Reduction in the likelihood of infecting others | 40% | [[15](#_ENREF_15),[16](#_ENREF_16)] |
| Threshold of infection risk | 0-100% | Health behavior survey (See S2) |
| Threshold of social standard | 0-100% | Health behavior survey |

*. The table is adopted from previoius work in [[12](#_ENREF_12)]
