# Supplementary material for: Evaluating the Combined Effectiveness of Influenza Control Strategies and Human Preventive Behavior
Source: PLoS One. 2011 Oct 17;6(10):e24706. doi: 10.1371/journal.pone.0024706 (PMC3197180; doi:10.1371/journal.pone.0024706)
Supplement: Text S1 — (DOC) [file pone.0024706.s006.doc]

**Supplementary Information**

**Evaluating combined effectiveness of influenza control strategies and human preventive behavior**

Liang Mao (Corresponding author)

[liangmao@ufl.edu](mailto:liangmao@ufl.edu)

Department of Geography, University of Florida, Gainesville, Florida, 32601, United States

**S1 Simulation Model**

**S1.1 Simulation of contact network**

The diffusions of influenza disease and human preventive behavior are inevitably related to human contacts. In the previous work, an agent-based model was established to simulate a contact network in the study area . The network consists of 985,001 discrete individuals as modeling agents, and daily contacts between individuals. Individual contacts are assumed to take place during three time periods in a day and at four types of locations. The three time periods include: the daytime, pastime and nighttime, while the four types of locations refer to homes, workplaces (schools, financial offices, administrative units, industrial factories, etc.), service places (utility companies, health facilities, grocery stores, etc.), and neighbor households (households located in the same census block group). Individuals travel between time and location, and have contact with different groups of individuals, such as family members, co-workers, clerks, and friends. These spatial-temporally varying contacts link all individuals into a city-wide network, which provides a basis for influenza transmission and behavioral dispersion.

The contact network was established by simulating three populations of daytime, nighttime and pastime, respectively, and linking them together based on the census data, business data, road network data, and travel survey statistics (Figure S1) . The census block group data was used to construct a nighttime population of individuals and households that matches the age and household structure of the real study area. The nighttime population is geo-referenced to household locations, expressed by (*x, y*) coordinates, according to the centroids of residential land parcels . Then, the information of nighttime population was used to generate the daytime population at workplaces. The nighttime population was assigned to workplaces based on the travel time to work (from census data), industrial codes (types) of businesses, and the road network through which individuals travel to work. The assignment to workplaces completed the daytime population and linked this population to the nighttime population at homes. Next, the pastime population was generated according to the information of previous two populations and a regional travel survey . Based on the frequency statistics in the travel survey, individuals were assigned a number of service trips, associated with the service type, travel mode and duration of each trip. Individuals were allocated to neighbor households nearby and service-oriented places (8,109 out of 36,839 businesses based on industrial codes) to complete the pastime population. In such a way, the pastime population is linked to the other two populations, and the entire contact network of the study area had been established. Detailed algorithms to generate these three populations have been reported previously .

**S1.2 Influenza-only model**

The influenza-only model has been previously established in the study area for studying influenza spread patterns . This is an agent-based stochastic model with discrete time steps and explicit spatial representation. The transmission process was simulated by repeatedly tracing susceptible contacts of infectious individuals through the modeled network, and identifying who would receive the infection in the next time step. The receipt of infection was modeled as a stochastic event determined by the age groups of receivers and the infectivity of viral strains, which was expressed by a disease parameter *R_0_*. Theoretically, *R_0_* (called the basic productive number) is defined as the number of secondary cases caused by a single infected case in a wholly susceptible population. Each modeled individual can take one of four infection status, i.e., susceptible, latent, infectious, or recovered. The progress of infection status follows the natural history of influenza, including the latent, incubation, and infectious periods. Ultimately, infectious individuals recover from influenza, and develop immunity to future transmission events. The major model parameters for simulating influenza transmission are listed in Table S1. Particularly for *R_0_*, this research tested a range of probabilities of infection in simulation and identified which value produce a simulated *R_0_* that fits the empirical observations (Table S1). The simulated *R_0_* was calculated by tracing the number of infections directly caused by the index cases in each model realization, and then averaged by 50 realizations.

To initialize influenza transmission through the modeled contact network, five infectious individuals are randomly seeded into the study area at the first day of simulation. The Monte-Carlo method was used to randomize the five infective seeds, daily contacts of individuals, the infections among these contacts and the development of symptoms. The simulation takes a tri-daily time step and lasts for 150 days.

**S1.3 Dual-diffusion model**

To further account for individual preventive behavior, this research employs an agent-based ‘dual-diffusion’ model that simulates the simultaneous diffusion of influenza and individual preventive behavior during an epidemic. These two diffusion processes interact with one another, i.e., the diffusion of influenza motivates the propagation of preventive behavior, which in turn limits the influenza diffusion. The diffusion of influenza is simulated with the same parameter settings of the influenza-only model aforementioned (Table S1). The diffusion of individual preventive behavior is propelled by two types of interpersonal influence, i.e., the perceived infection risk and the perceived social standard. The former was represented as the proportion of influenza cases among an individual’s contacts, while the latter was measured by the proportion of behavioral adopters among an individual’s contacts . Individuals are simulated to perceive these two proportions through the contact network every day. Once either proportion exceeds an individualized threshold, an individual will be convinced to adopt and practice preventive behavior .

To formulate these two threshold effects, each modeled individual is associated with three attributes and two actions. The three attributes involve an adoption status (*adopter* or *non-adopter*), the threshold of infection risk, and the threshold of social standard . All individuals are intially set to be non-adopters. The two threshold attributes are estimated using a health behavior survey conducted in 2010 (details are provided in the following section). To fit the statistical distributions from the survey (Figure S2 and S3), the Monte-Carlo simulation method is used to assign threshold values to individuals. The two actions are individuals’ evaluation of infection risk and social standard, respectively. These two actions reflect inter-personal influences between individuals, and either action can alter the adoption status of individuals. Once individuals adopt preventive behavior, the likelihood of being infected or infecting others will be reduced. To quantify this reductive effect, the use of antiviral drugs is taken as an example of preventive behavior in the simulation. This is because its efficacy on influenza infectivity is more conclusive than other behavior, such as washing hands and wearing facemasks. The major parameters for simulating preventive behavior are shown in Table S2. Similar to the influenza-only model, five infectious individuals are randomly seeded into the study area at the first day of simulation, and the simulation takes a tri-daily time step and lasts for 150 days.

**S2 Health behavior survey**

The health behavior survey was conducted among adult residents of the study area. The survey had been approved by the Social and Behavioral Sciences Institutional Review Board, University at Buffalo, State University of New York. The waiver of informed consent was obtained from the university review board for this research. An on-line survey and a postal mail survey were carried out to recruit participants. An electronic questionnaire was posted on a professional survey website for one month, while hard copies of questionnaire were simultaneously mailed to 500 residents (50 zip-code areas × 10 residents per area). Besides age group and gender, survey participants were invited to answer two questions:

1) *Suppose you have 10 close contacts, including household members, colleagues, and close friends, when how many of them GET INFLUENZA may you consider using anti-viral flu drugs?*

2) *Suppose you have 10 close contacts, including household members, colleagues, and close friends, when how many of them start to USE ANTIVIRAL DRUGS may you consider using anti-viral drugs yourself?*

A total of 381 respondents participated in the survey, including 262 respondents in the online survey (out of 273 visitors, a 96% response rate) and 119 in the postal mail survey (out of 500 questionnaires, a 24% response rate) .Based on the answers, the thresholds of perceived infection risks and adoption pressure are estimated by gender. Figure S2 shows the probability distributions of thresholds of infection risks in male and female population respectively. The distributions for the two genders are very similar. For both male and female, about half of the respondents would not take antiviral drugs unless they are ill. Their adoptive behavior is not influenced by surrounding sick people. The other half of respondents may adopt the antiviral drugs when less than 5 out of 10 contacts are infected. Figure S3 indicates the probability distributions of thresholds of adoption pressure by gender. Similarly, about a half of respondents are not willing to adopt, no matter how many their contacts have adopted. For the other half respondents, most of them may be convinced to adopt when 10% - 60% of their contacts have adopted.

**References**

1. Mao L, Bian L (2010) Spatial-temporal transmission of influenza and its health risks in an urbanized area. Computers, Environment and Urban Systems 34: 204-215.

2. Bian L (2004) A conceptual framework for an individual-based spatially explicit epidemiological model. Environment and Planning B 31: 381-395.

3. NYS-GIS-Clearinghouse (2009) Parcel Data by Town, City, and Village Albany, NY: NYS GIS Clearinghouse.

4. GBNRTC (2002) Greater Buffalo-Niagara Regional Transportation Council 2002 Regional Transportation Survey Results Report. Buffalo, NY.

5. Diekmann O, Heesterbeek JAP, Metz JAJ (1990) On the definition and the computation of the basic reproduction ratio R_0_ in models for infectious diseases in heterogeneous populations. Journal of Mathematical Biology 28: 365-382.

6. Mills CE, Robins JM, Lipsitch M (2004) Transmissibility of 1918 pandemic influenza. Nature 432: 904-906.

7. Cauchemez S, Valleron AJ, Boelle PY, Flahault A, Ferguson NM (2008) Estimating the impact of school closure on influenza transmission from Sentinel data. Nature 452: 750-754.

8. Heymann DL (2004) Control of Communicable Diseases Manual. Washington, DC: American Public Health Association.

9. Halloran ME, Ferguson NM, Eubank S, Ira M. Longini J, Cummings DAT, et al. (2008) Modeling targeted layered containment of an influenza pandemic in the United States. Proceedings of the National Academy of Sciences 105: 4639-4644.

10. CDC (2008) Key Facts About Seasonal Influenza (Flu). Atlanta: Center for Disease Control and Prevention.

11. Ferguson NM, Cummings DA, Fraser C, Cajka JC, Cooley PC, et al. (2006) Strategies for mitigating an influenza pandemic. Nature 442: 448-452.

12. Mao L, Bian L (2011) Agent-based Simulation for a Dual-Diffusion Process of Influenza and Human Preventive Behavior. International Journal of Geographical Information Science In press.

13. Granovetter M (1978) Threshold models of collective behavior. American journal of sociology 83: 1420-1443.

14. Valente TW (1996) Social network thresholds in the diffusion of innovations. Social Networks 18: 69-89.

15. Hayden FG (2001) Perspectives on antiviral use during pandemic influenza. Philosophical Transactions of the Royal Society of London Series B 356: 1877-1884.

16. Longini IM, Nizam A, Xu S, Ungchusak K, Hanshaoworakul W, et al. (2005) Containing pandemic influenza at the source. Science 309: 1083-1087.
